# Surgery combined with antibiotics for the treatment of endogenous endophthalmitis caused by liver abscess

**DOI:** 10.1186/s12879-020-05390-z

**Published:** 2020-09-07

**Authors:** Yue Wang, Xue Wang, Yu Di

**Affiliations:** Department of Ophthalmology, Shengjing Hospital of China Medical University, Heping district, Sanhao Road 36, Shenyang, 110004 People’s Republic of China

**Keywords:** Endogenous endophthalmitis, *Klebsiella pneumoniae*, Liver abscess, Vitrectomy, Visual acuity

## Abstract

**Backgrounds:**

Endogenous endophthalmitis is a serious disease caused by intraocular infection that can rapidly progress to cause blindness. This study evaluated the clinical features, surgical and antibiotics treatment strategies, and treatment outcomes in patients with endophthalmitis caused by liver abscess.

**Methods:**

Between April 2014 and April 2019, the clinical data of 16 patients (19 eyes) with endophthalmitis associated with liver abscess who underwent surgery at Shengjing Hospital were retrospectively analyzed. Furthermore, we evaluated the final visual outcomes in the patients to determine the efficacy of surgery.

**Results:**

Fifteen patients (18 eyes) underwent intravitreal injection followed by vitrectomy after admission. One patient (1 eye) only underwent intravitreal injection. Of the 16 patients, 3 patients (3 eyes) had recurrent intraocular inflammation and eventually underwent evisceration. Systemic antibiotics were administered for all patients based on the results of vitreous humor culture, blood culture, and antibiotic susceptibility tests. Outpatient follow-ups were performed until the patients were stable (6 months). Of the 19 eyes, 1 eye (5%) had visual acuity restored to 20/200, 6 eyes (31%) had visual acuity restored to counting fingers (CF), 2 eyes (11%) had visual acuity restored to hand motion (HM), 4 eyes (22%) showed only light perception (LP), and the remaining 6 eyes (31%) showed no light perception (NLP). Drug susceptibility tests suggested that the carbapenems exhibited significant effects in the inflammatory reaction.

**Conclusion:**

Endogenous endophthalmitis caused by liver abscess is a very serious condition, and the final visual outcome is poor. Timely surgical intervention combined with antibiotic treatment is essential, and the primary disease must be treated to control disease progression at the earliest.

## Background

Endophthalmitis is an ocular disease that can lead to serious visual acuity damage [1, 2]. Endogenous endophthalmitis as a result of liver abscess is caused by the hematogenous spread of pathogenic bacteria from the liver abscess, which passes through the blood–retinal barrier, leading to infection of the eye [3]. In recent years, many cases have been reported in Asia, with *Klebsiella pneumoniae* being the most common causative agent [4, 5]. Since the disease is relatively occult during the early stage, it can rapidly infect intraocular tissues, resulting in irreversible damage to the photoreceptor cells in the retina. This causes irreversible damage to the eye, leading to a significant impact on visual function, and can also potentially be life-threatening [6–8]. Therefore, prompt management during the early stage is essential to prevent permanent blindness and mortality. In this study, we evaluated the clinical features, surgical strategies, pathogenic bacterial features, and visual outcomes in patients with endophthalmitis caused by liver abscess.

## Methods

### Study design and clinical data collection

We retrospectively analyzed patients with liver abscess–associated endophthalmitis who were admitted to the Department of Ophthalmology of Shengjing Hospital of China Medical University between April 2014 and April 2019. Patients with the following characteristics were included: diagnosis of endophthalmitis by operation and examination and liver abscess confirmed by imaging. Patients with the following risk factors were excluded: history of keratitis, history of intraocular surgery, trauma, or glaucoma [9]. Finally, 16 patients (19 eyes) of 72 patients with endogenous endophthalmitis were included in the study. Clinical data collected included patient demographic information, length of hospitalization, department admitted after initial diagnosis, medical history, patient symptoms, treatment strategy, and degree of visual acuity correction after surgery. All procedures involving human participants were conducted in accordance with the tenets of the Declaration of Helsinki. The study design was approved by the Ethics Committee of the Department of Ophthalmology of Shengjing Hospital.

### Treatment strategy

Once a diagnosis of endogenous endophthalmitis was confirmed by ocular ultrasonography and fundus examination in patients who visited the ophthalmology clinic or after consultation with the Department of Hepatobiliary Surgery, the patients were treated through surgery. Patients either underwent vitrectomy using silicone oil combined with an intravitreal injection of antibiotics, or received an intravitreal injection alone. Of the 19 eyes, Only 1 patient (1 eye) was intravitreal injection alone. Depending on the treatment strategy and the surgeon’s level of experience, patients received an intravitreal injection of 1 mg/0.1 ml *vancomycin*, 2.25 mg/0.1 ml *ceftazidime*, and 0.2 mg/0.1 ml *dexamethasone*. *Tobramycin* and *dexamethasone* were administered as topical eye drops. The administration of systemic antibiotics was adjusted based on the results of the antibiotic susceptibility tests. Outpatient follow-ups were performed for 6 months, until the patients were stable.

### Statistical analysis

The visual acuity was recorded using logMAR as follows: counting fingers (CF) = 1.7 logMAR, hand motion (HM) = 2.0 logMAR, light perception (LP) = 2.3 logMAR, no light perception (NLP) = 3.0 logMAR [10, 11]. The visual acuity of patients who underwent evisceration was considered NLP. All data were analyzed using SPSS17.0. Statistical differences between preoperative and postoperative visual acuities were analyzed using Single sample Wilcoxon test, with a significance level set at *P* < 0.05.

## Results

### Baseline patient characteristics

Of the 16 patients enrolled in this study, the mean age was 56 years (40–67 years). There were 9 (56%) male patients and 7 (44%) female patients. Among the 19 eyes investigated, 10 eyes (53%) were right eyes, and 9 eyes (47%) were left eyes. The mean length of hospital stay was 18.75 days (6–37 days). Nine patients (56%) had an initial diagnosis of endophthalmitis. Six patients (38%) had liver abscess and 1 patient (6%) had fever as initial symptom. Significant systemic medical problems included hypertension in 6 patients (38%) and diabetes mellitus in 9 patients (56%). The characteristics are listed in Table 1.

**Table 1:** Characteristics of the patients

Characteristics	Values
Age (years)	56 (40–67)
Gender	
Male (n,%)	9 (56%)
Female (n,%)	7 (44%)
Left/right side	
Right (n,%)	10 (53%)
Left (n,%)	9 (47%)
Length of hospital stay (days)	18 (6 to 37)
Initial diagnosis	
Endophthalmitis (n,%)	9 (56%)
Liver abscess (n,%)	6 (38%)
Fever (n,%)	1 (6%)
Hypertension	6 (38%)
Diabetes	9 (56%)

### Liver abscess clinical characteristics

**Fig. 1:**
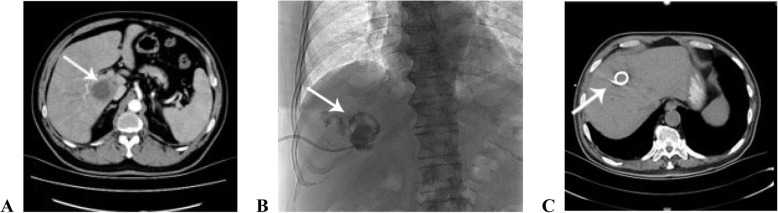
Clinical features of liver abscess. **a** Contrast-enhanced abdominal CT image of liver abscess suggestive of intrahepatic abscess. **b** Plain abdominal radiograph of the liver abscess after drainage, showing that the drainage catheter was well-positioned, and the abscess had subsided. **c** Contrast-enhanced abdominal CT image of the liver abscess after drainage, showing that the drainage catheter was well-positioned, and the abscess had subsided

Patients with a liver abscess displayed notable fever and upper right abdominal pain. An emergency blood test revealed a significant elevation of white blood cells and neutrophils, and C-reactive protein levels tested at the emergency department were also significantly elevated. Abdominal CT showed low-density lesions at various locations in the liver, which were suggestive of an intrahepatic abscess (Fig. 1a). The diameter of the liver abscess was 2.1–8.8 cm, whereas the average diameter was (4.17 ± 2.06) cm.

All patients underwent percutaneous liver abscess drainage, and received a follow-up plain abdominal radiography (Fig. 1b) and contrast-enhanced abdominal CT (Fig. 1c), both of which showed that the drainage catheter was well-positioned and the abscess had subsided.

### Clinical features of endophthalmitis

**Fig. 2:**
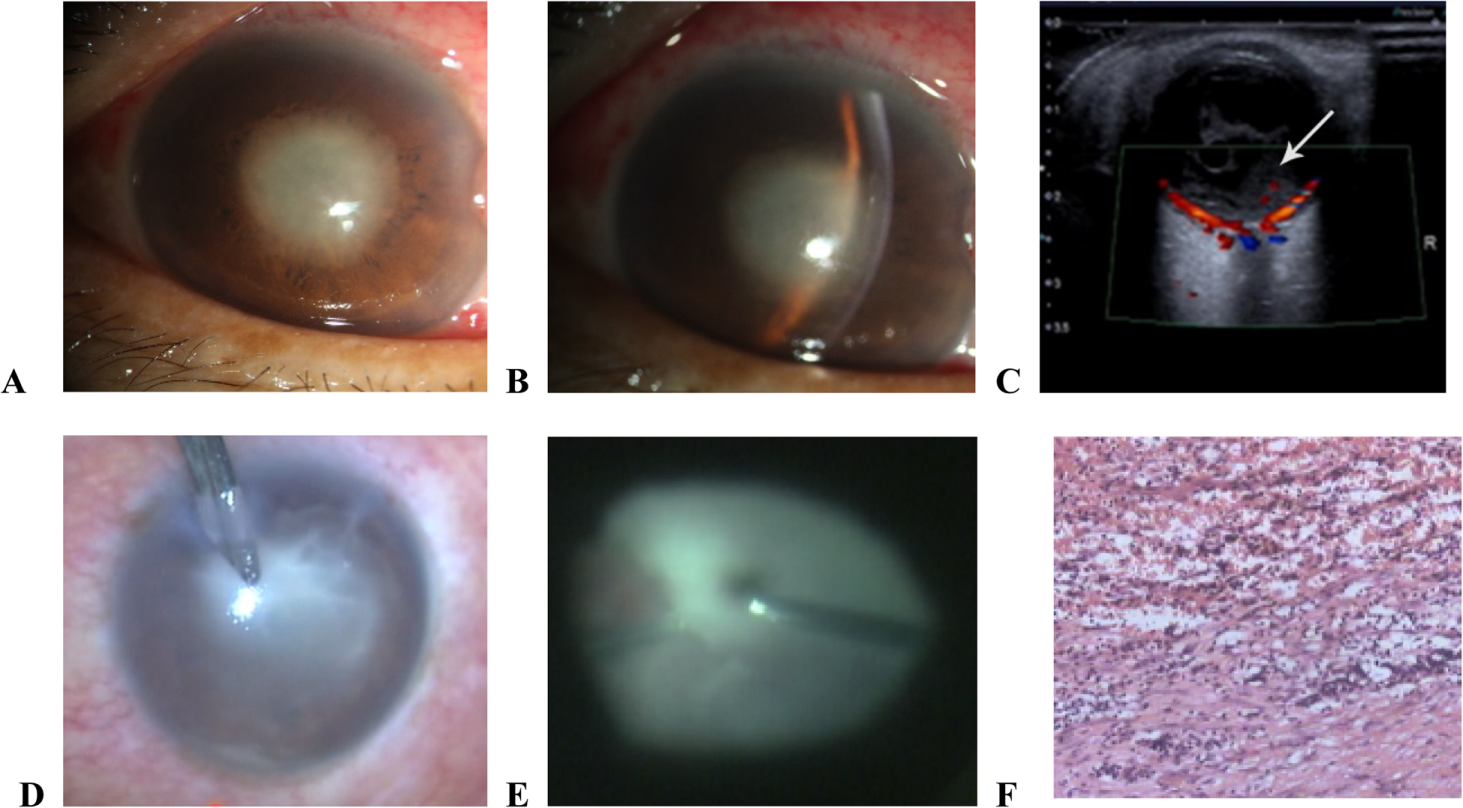
Clinical features of liver endophthalmitis. **a** and **b** Biomicroscopic examination revealing conjunctival hyperemia, corneal edema, and inflammation of the anterior chamber, accompanied by discernable pus accumulation or fibrinous exudates. **c** Three-dimensional ultrasound imaging of the eye showing turbidity in the form of streaks, dots, or floccules in the vitreous humor. **d**-**e** Purulent exudate was observed in the anterior chamber and vitreous humor. Tight adhesion of fibrinous exudate was observed in the vitreous cavity. **f** Pathological image of eye biopsy showing extensive neutrophil infiltration and piecemeal necrosis

Slit-lamp bio-microscopic examination revealed conjunctival hyperemia, corneal edema, and inflammation of the anterior chamber, accompanied by discernable pus accumulation or fibrinous exudates (Fig. 2a and b). Funduscopic examination did not form a sharp image of the retina. Three-dimensional ultrasound imaging of the eye showed turbidity in the form of streaks, dots, or floccules in the vitreous humor, which were suggestive of endophthalmitis (Fig. 2c). During the vitrectomy, pus accumulation in the anterior chamber and vitreous humor was observed, and a large amount of purulent exudate was present in the vitreous cavity (Fig. 2d-e). During vitrectomy, it was observed that the retina was light in color, and the fibrinous exudate was tightly adherent. An eye biopsy for patients who underwent evisceration showed that the eye was partially lined with squamous epithelium, with infiltration of neutrophils cells underneath. The lining contralateral to the squamous epithelium showed extensive neutrophil infiltration and piecemeal necrosis (Fig. 2f).

### Microbial culture

Microbial cultures were performed on the drainage fluid, blood, and vitreous humor of all patients. *Klebsiella pneumoniae* was identified in 8 (50%) patients, *Serratia marcescens* in 1 (6%) patient, *Bacillus licheniformis* in 1 (6%) patient, and *Staphylococcus warneri* in 1 (6%) patient. For the remaining 5 (32%) patients, no apparent bacteria or fungus could be identified in culture. Results of the postoperative blood and drug susceptibility tests suggested that the carbapenems, such as *Imipenem* and *Meropenem,* exhibited significant effects in the inflammatory reaction. The results are shown in Table 2.

**Table 2:** Culture and drug sensitivity tests of endogenous endophthalmitis patients

Patient	Pathogenic bacteria	Culture	Antibiotic resistance
		V/ B/ D	
1	*Klebsiella pneumoniae*	+/−/NA	AMP, CFZ, TE, SSS
2	*Klebsiella pneumoniae*	+/+/NA	AMP, AZT, CIP, CRO, FEP, CFZ, NIT, LVX
3	*Klebsiella pneumoniae*	+/−/NA	AMP
4	*Klebsiella pneumoniae*	+/−/NA	CTT, NIT, CFZ,
5	*Klebsiella pneumoniae*	+/−/NA	AMP
6	*Klebsiella pneumoniae*	+/−/NA	AMP, TE
7	*Klebsiella pneumoniae*	+/−/+	AMP
8	*Klebsiella pneumoniae*	NA/−/+	NA
9	*Serratia marcescens*	+/+/+	CTT, NIT, CFZ, SSS
10	*Bacillus licheniformis*	NA/−/+	NA
11	*Staphylococcus warneri*	NA/−/+	NA

*V* Vitreous; *B* Blood; *D* drainage fluid; *NA* Not available; *AMP* Ampicillin; *CFZ* Cefazolin; *TE* Tetracycline; *SSS* Sulfonamides; *AZT* Aztreonam; *CIP* Ciprofloxacin; *NIT* Nitrofurantoin; *CTT* Cefotetan; *FEP* Cefepime; *CRO* Ceftriaxone; *LVX* Levofloxacin

### Restoration of visual activity

Sixteen eyes received ophthalmologic surgery for treatment for primary disease, combing with postoperative intravenous antibiotics, which effectively controlled inflammation. Three eyes had recurrent inflammation, and evisceration was subsequently performed. One eye (5%) had visual acuity restored to 20/200, 6 eyes (31%) had visual acuity restored to CF, 2 eyes (11%) had visual acuity restored to HM, 4 eyes (22%) showed only LP, and the remaining 6 eyes (31%) eventually showed NLP. Single sample Wilcoxon test to compare the preoperative and postoperative visual acuities to determine treatment efficacy showed a statistically significant difference (*z = − 2.598, P < 0.05*). The results are shown in Table 3.

**Table 3:** Visual acuity of the endogenous endophthalmitis patients

Visual acuity	Initial visual activity	Final visual outcome
Better than 20/200	1 (5%)	1 (5%)
Counting fingers (CF)	0 (0)	6 (31%)
Hand motion (HM)	3 (16%)	2 (11%)
Light perception (+) (LP)	10 (53%)	4 (22%)
Light perception (−) (NLP)	5 (26%)	6 (31%)
LogMAR Median (IQR)	2.3 (2.3–3.0)	2.3 (1.7–3.0)

## Discussion

Recent studies have shown that endophthalmitis caused by liver abscess accounts for an increasing percentage of endogenous endophthalmitis cases each year, particularly in East Asia [12]. A study on endogenous endophthalmitis conducted in Korea showed that 25% of primary lesions were liver abscesses [13], whereas a study in Taiwan found that 53% of primary lesions were liver abscesses, and 61% of cases were caused by *Klebsiella pneumoniae,* and showed the trend of drug resistance [14]. In the present study, microbial cultures of the vitreous humor, as well as blood cultures were obtained from all patients. Bacteria could be identified in 11 (69%) cases, 8 (50%) of which were positive for *Klebsiella pneumoniae*. In Asia, the incidence rate of multidrug-resistant and hypervirulent *Klebsiella pneumoniae* strains increased [15]. We found that there were 3 patients with carbapenem resistance; however, the patients were not included in our study because of the surgery and the discontinued visiting of the patients.

One patient’s endophthalmitis was caused by *Serratia marcescens*, and the final visual outcome of the 2 eyes was NLP. Endophthalmitis caused by *Serratia marcescens* is very rare, where most of the cases progress to NLP. Most of the reported cases have a history of interventional surgery, oral surgery, and intravenous drug use [16]. Both liver abscess and endophthalmitis caused by *Staphylococcus walleriae* and *Bacillus licheniformis* are uncommon, implying they should be paid more attention in the clinics. The ideal condition for performing a bacterial culture is during the absence of antibiotic treatment following disease onset. However, due to the rapid progression of the disease, and the lack of a standardized antibiotic treatment strategy for endogenous endophthalmitis, it is difficult to control this condition, and the results of the culture may be unreliable [17]. Therefore, for effective management of endogenous endophthalmitis, blood samples should be collected before starting antibiotic treatment [18].

Liver abscess–associated endophthalmitis can significantly impact the visual acuity of patients, and has a poor prognosis. A study conducted in Southern California investigating endophthalmitis caused by *Klebsiella pneumoniae* showed that approximately half of the patients required enucleation [19]. It has been reported that early use of antibiotics combined with timely vitrectomy in patients with endophthalmitis can effectively improve the visual acuity [20]. In the present study, we found that while the condition of the patients with liver abscess–associated endophthalmitis could be improved by vitrectomy combined with intravitreal injection, the overall prognosis was poor. Endogenous endophthalmitis caused by liver abscess is a metastatic infection, in which inflammation occurs rapidly and pathogenic bacteria can invade the inner eyes in a short period of time, which makes surgery more difficult [2]. In this study, 56% (9 patients) with endogenous endophthalmitis were initially diagnosed with endophthalmitis, other 44% (7 patients) were only a liver abscess and fever. Eye symptoms are easily neglected when rescuing critical symptoms and focusing on systemic conditions, thereby missing the opportunity for early diagnosis and effective treatment. Furthermore, the clinical manifestations of liver abscess–associated endophthalmitis are nonspecific, and the condition can easily be misdiagnosed as other diseases, such as uveitis, leading to a delay in diagnosis and treatment. Four patients had NLP vision when they were transferred to an ophthalmology department, and their vision could not be preserved. Therefore, when treating patients with severe liver abscess, especially those in coma, doctors should pay attention to the patients’ eyes.

Vitrectomy is effective for the treatment of retinal detachment, vitreous hemorrhage, and diabetic retinopathy. It is also the most commonly used surgical method that has demonstrated a definitive efficacy for the treatment of endophthalmitis [21–23]. Vitrectomy can remove intravitreal inflammatory lesions, bacteria and toxins, and reduce the damage caused by the effect of toxic substances on the retinal function [24]. It may also rescue vitreous transparency and reduce or avoid tractional retinal detachment [25, 26]. Intravitreal injection enables the rapid and effective delivery of antibiotics, to achieve high local drug concentrations. This allows the antibiotics to target the pathogenic bacteria more effectively, thereby inhibiting bacterial growth and controlling inflammation. Concurrent intravitreal vancomycin, ceftazidime and dexamethasone are recommended for patients with infectious endophthalmitis caused by pyogenic liver abscess [27, 28].According to experimental and clinical observations, most clinicians believe that injection of *vancomycin, ceftazidime* and *dexamethasone* into the vitreous cavity is safe and doses not lead to retinal toxicity. Studies have shown that intravitreal antibiotic injection for susceptible pathogens within 48 h may help in retaining the visual acuity in some patients with liver abscess–associated endophthalmitis, and patients who received antibiotic intervention 48 h following the disease onset ultimately had poorer visual acuity [29]. Despite prompt treatment, the visual sequelae of endogenous endophthalmitis caused by liver abscess are frequent and outcomes are poor. In the current series of studies, only 6 patients (31%) achieved CF or better visual acuity at 6 months of follow-up. In addition, because some patients who underwent vitrectomy, are accompanied with bacteremia, postoperative infection is still a high-risk factor for blindness. Three patients in this study underwent enucleation as a result of the infection not effectively controlled in time. Repeated intravitreal and periocular injections of antibiotics and dexamethasone could prevent enucleation [30].Therefore, it is necessary to inject antibiotics intravenously according to the results of drug sensitivity after vitrectomy.

The present study showed that upon diagnosis of endophthalmitis caused by liver abscess, treatment should be initiated immediately and include intravitreal antibiotics injections, and vitrectomy. We concluded that vitrectomy combined with intravitreal injection is effective in the treatment of endogenous endophthalmitis. Further, the active treatment of the primary lesions was critical. The administration of systemic antibiotics should be accompanied by active treatment of surgical abscess drainage. Antibiotics should be selected according to the results of drug sensitivity. Cephalosporins or carbapenems can be selected according to experience before obtaining drug sensitivity results [30, 31].Therefore, The disease was effectively mitigated, and progression to panophthalmitis was controlled.

Our study had some limitations. Firstly, the limited case data because of the retrospective nature of the study, secondly, the sample size of this study was small. Future studies may include a larger sample size and a multicentered approach.

## Conclusion

Endogenous endophthalmitis caused by liver abscess is a serious disease with rapid progression and poor prognosis. In this study, early intravitreal injection and vitrectomy are important for improving visual acuity. Further, the systematic and thorough treatment of liver abscess is of great significance in preventing the recurrence of endophthalmitis because of liver abscess. Additionally, we suggest that surgeons should pay attention to the symptoms of the eyes while diagnosing and treating patients with liver abscess.

## Data Availability

All data generated or analyzed during this study are included in this published article (and its supplementary information files).

## Abbreviations

CF: Counting fingers
HM: Hand motion
LP: Light perception
NLP: No light perception
